# Acid Ceramidase Rescues Cystic Fibrosis Mice from Pulmonary Infections

**DOI:** 10.1128/IAI.00677-20

**Published:** 2021-01-19

**Authors:** Katrin Anne Becker, Rabea Verhaegh, Hedda-Luise Verhasselt, Simone Keitsch, Matthias Soddemann, Barbara Wilker, Gregory C. Wilson, Jan Buer, Syed A. Ahmad, Michael J. Edwards, Erich Gulbins

**Affiliations:** a Institute of Molecular Biology, University of Duisburg-Essen, Essen, Germany; b Institute of Medical Microbiology, Medical School Essen, University Hospital Essen, University of Duisburg-Essen, Essen, Germany; c Department of Surgery, University of Cincinnati Medical School, Cincinnati, Ohio, USA; Georgia Institute of Technology School of Biological Sciences

**Keywords:** acid ceramidase, ceramide, sphingosine, *Pseudomonas aeruginosa*, cystic fibrosis, pneumonia

## Abstract

Previous studies have shown that sphingosine kills a variety of pathogenic bacteria, including Pseudomonas aeruginosa and Staphylococcus aureus. Sphingosine concentrations are decreased in airway epithelial cells of cystic fibrosis (CF) mice, and this defect has been linked to the infection susceptibility of these mice. Here, we tested whether the genetic overexpression of acid ceramidase rescues cystic fibrosis mice from pulmonary infections with P. aeruginosa. We demonstrate that the transgenic overexpression of acid ceramidase in CF mice corresponds to the overexpression of acid ceramidase in bronchial and tracheal epithelial cells and normalizes ceramide and sphingosine levels in bronchial and tracheal epithelial cells. In addition, the expression of β1-integrin, which is ectopically expressed on the luminal surface of airway epithelial cells in cystic fibrosis mice, an alteration that is very important for mediating pulmonary P. aeruginosa infections in cystic fibrosis, is normalized in cystic fibrosis airways upon the overexpression of acid ceramidase. Most importantly, the overexpression of acid ceramidase protects cystic fibrosis mice from pulmonary P. aeruginosa infections. Infection of CF mice or CF mice that inhaled sphingosine with P. aeruginosa or a P. aeruginosa mutant that is resistant to sphingosine indicates that sphingosine and not a metabolite kills P. aeruginosa upon pulmonary infection. These studies further support the use of acid ceramidase and its metabolite sphingosine as potential treatments of cystic fibrosis.

## Abstract (precis)

Previous studies have shown that sphingosine kills a variety of pathogenic bacteria, including Pseudomonas aeruginosa and Staphylococcus aureus. Sphingosine concentrations are decreased in airway epithelial cells of cystic fibrosis (CF) mice, and this defect has been linked to the infection susceptibility of these mice. Here, we tested whether the genetic overexpression of acid ceramidase rescues cystic fibrosis mice from pulmonary infections with P. aeruginosa.

## INTRODUCTION

Cystic fibrosis (CF) is caused by mutations of the CF transmembrane conductance regulator gene (human, *CFTR*; mouse, *Cftr*). Mutations of CFTR are relatively common, with a frequency of approximately 1 in 50 people in the European Union and the United States. Thus, cystic fibrosis is the most common autosomal recessive disorder, affecting 1 of every 2,500 children born in Western countries. Cystic fibrosis is characterized by several respiratory, reproductive, and gastrointestinal symptoms. However, at present, chronic pulmonary colonization with bacterial pathogens, in particular Pseudomonas aeruginosa, and the development of chronic pneumonia, chronic inflammation, and progressive pulmonary fibrosis are the primary causes of morbidity and mortality of these patients (1).

The molecular mechanisms that mediate the three hallmarks of the disease, i.e., infection, inflammation, and fibrosis, still require further definition.

CFTR functions as an epithelial chloride channel, and it has been suggested that altered water absorption may change the composition of the mucus present on the epithelial cells of the respiratory tract in cystic fibrosis lungs, resulting in reductions of mucociliary clearance, the function of defensins, and the ability to eliminate P. aeruginosa (2). However, the concept of altered mucociliary clearance as a cause of increased infection susceptibility was difficult to prove *in vivo* (3).

We and others have recently reported that the lipid composition of CF airway epithelial cells is significantly altered, with an increase of ceramide and a decrease of sphingosine in the luminal membrane of tracheal and bronchial epithelial cells (4–16). We have previously shown that sphingosine is central for killing bacterial pathogens in cystic fibrosis airways and that the decrease of sphingosine in cystic fibrosis airway epithelial cells is central for the increased infection susceptibility of cystic fibrosis mice or human cells isolated from cystic fibrosis patients (11–13). We and others also demonstrated that sphingosine kills a variety of bacterial species *in vitro* and *in vivo*, including P. aeruginosa, Escherichia coli, Haemophilus influenzae, Moraxella catarrhalis, Burkholderia cepacia, Staphylococcus aureus, Acinetobacter baumannii, and Porphyromonas gingivalis (11, 12, 17–20). Luminal ceramide concentrations are very low in healthy epithelial cells of the airways and increased in cystic fibrosis epithelial cells. This accumulation of ceramide results in the ectopic expression and clustering of β1-integrins that further support the infection susceptibility of cystic fibrosis cells (13).

Thus, the balance of ceramide and sphingosine plays a central role in bacterial infections of cystic fibrosis cells. The concentrations of the two lipids in airway epithelial cells seem to be mainly regulated by acid ceramidase, as indicated by studies applying acid ceramidase in the lungs of cystic fibrosis mice (11, 13), although the significance of endogenous acid ceramidase for bacterial infections of cystic fibrosis mice remains to be determined.

To analyze the role of acid ceramidase in cystic fibrosis, we generated mice that lack functional Cftr and overexpress acid ceramidase (mouse gene, *Asah1*). We then determined alterations of lipids in these mice and, most importantly, the infection susceptibility of these mice in comparison to Cftr-deficient mice. Using a sphingosine-resistant P. aeruginosa mutant, we further analyzed whether sphingosine or a metabolite mediates the killing of P. aeruginosa.

We demonstrate that the overexpression of acid ceramidase in cystic fibrosis mice normalizes ceramide and sphingosine concentrations in cystic fibrosis epithelial cells. This results in a normalization of the ectopic expression of β1-integrins in epithelial cells. Most importantly, the overexpression of acid ceramidase was sufficient to prevent pulmonary infections of cystic fibrosis mice with P. aeruginosa. Wild-type mice rapidly cleared wild-type P. aeruginosa upon pulmonary infection but were unable to eliminate a sphingosine-resistant P. aeruginosa strain. Cystic fibrosis mice were highly sensitive to pulmonary infection with the wild-type strain or a sphingosine-resistant mutant of P. aeruginosa. Inhalation of sphingosine restored the resistance of CF mice against wild-type P. aeruginosa strains but had no effect on the sphingosine-resistant P. aeruginosa mutant.

## RESULTS

**FIG 1:**
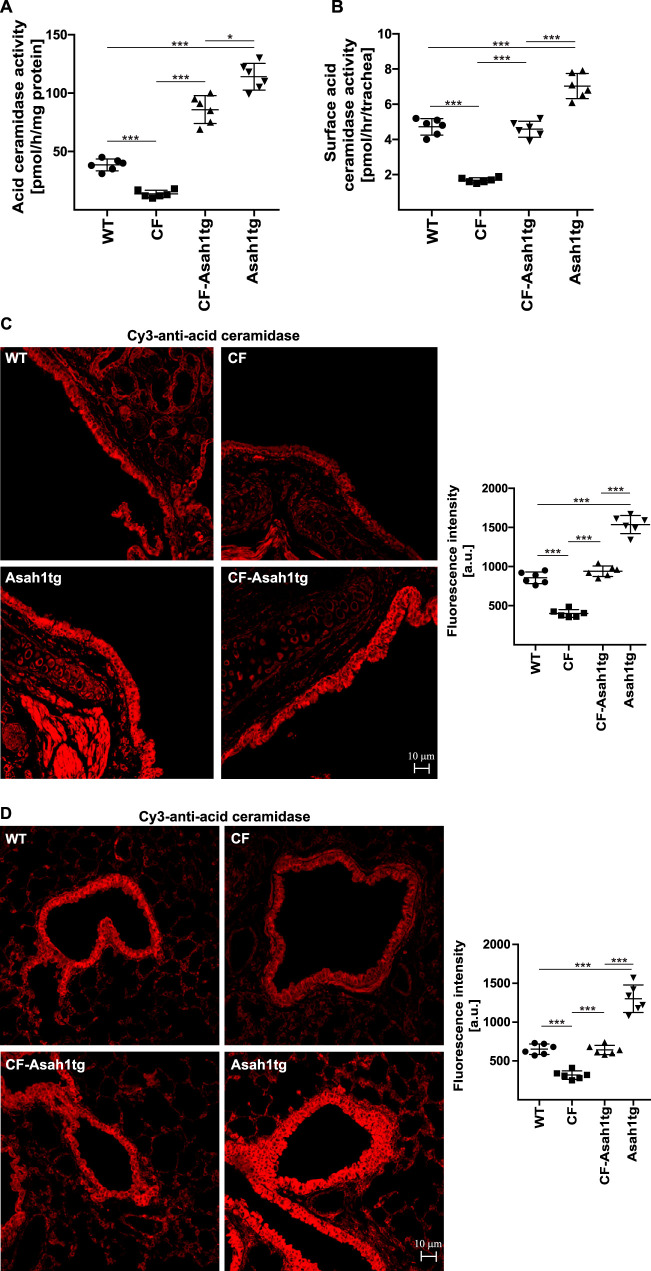
Transgenic expression of acid ceramidase in CF mice increases acid ceramidase activity in the trachea and lung. (A) Activity of acid ceramidase determined in tracheal extracts from syngeneic wild-type (WT), CF, Asah1tg, and CF-Asah1tg mice. Acid ceramidase activity is greatly reduced in CF mice and increased to wild-type levels in the trachea of CF mice that are transgenic for acid ceramidase (CF-Asah1tg). Shown are the means ± SD from 6 independent experiments. *, *P* < 0.05; ***, *P* < 0.001 (by ANOVA and *post hoc* Student’s *t* test). (B) The *in situ* surface activity of acid ceramidase in epithelial cells is decreased in CF epithelial cells and normalized upon transgenic expression of acid ceramidase. The activity of acid ceramidase in epithelial cells from syngeneic wild-type, CF, Asah1tg, and CF-Asah1tg mice was determined by measuring the consumption of [^14^C]ceramide in the trachea *in situ*. Given are the means ± SD from 6 independent experiments. ***, *P* < 0.001 (by ANOVA and *post hoc* Student’s *t* test). (C and D) Typical examples of confocal microscopy studies of the trachea (C) and lung (D) analyzing expression levels of acid ceramidase in epithelial cells. Confocal microscopy studies were performed with Cy3-coupled anti-acid ceramidase antibodies. The studies are representative of data from 6 samples with similar results. Fluorescence was also quantified. Given are the means ± SD (*n* = 6 each). ***, *P* < 0.001 (by ANOVA and *post hoc* Student’s *t* test). a.u., arbitrary units.

To define the role of acid ceramidase in airway epithelial cells for the increased infection susceptibility of cystic fibrosis mice, we crossed *Cftr*-deficient (CF) mice with acid ceramidase transgenic mice in order to overexpress acid ceramidase in cystic fibrosis mice. This novel mouse strain was named CF-Asah1tg. The activity of acid ceramidase in the trachea of CF-Asah1tg mice was higher than that in wild-type mice but lower than the activity of the acid ceramidase in control Asah1 transgenic mice (Fig. 1A). Syngeneic CF mice showed a reduced activity of the acid ceramidase in the trachea (Fig. 1A). More importantly, the activity of the acid ceramidase was also increased in tracheal epithelial cells of CF-Asah1tg mice compared to CF mice. The activity of the enzyme in CF tracheal epithelial cells of CF mice was reduced compared to that in wild-type controls (Fig. 1B). Note that the activity of the enzyme was determined *in situ* in tracheal epithelial cells (Fig. 1B). The activity measurements corresponded to the protein expression levels of the acid ceramidase in tracheal and bronchial epithelial cells, which were reduced in CF cells and restored in CF-Asah1tg mice compared to wild-type mice (Fig. 1C and D).

**FIG 2:**
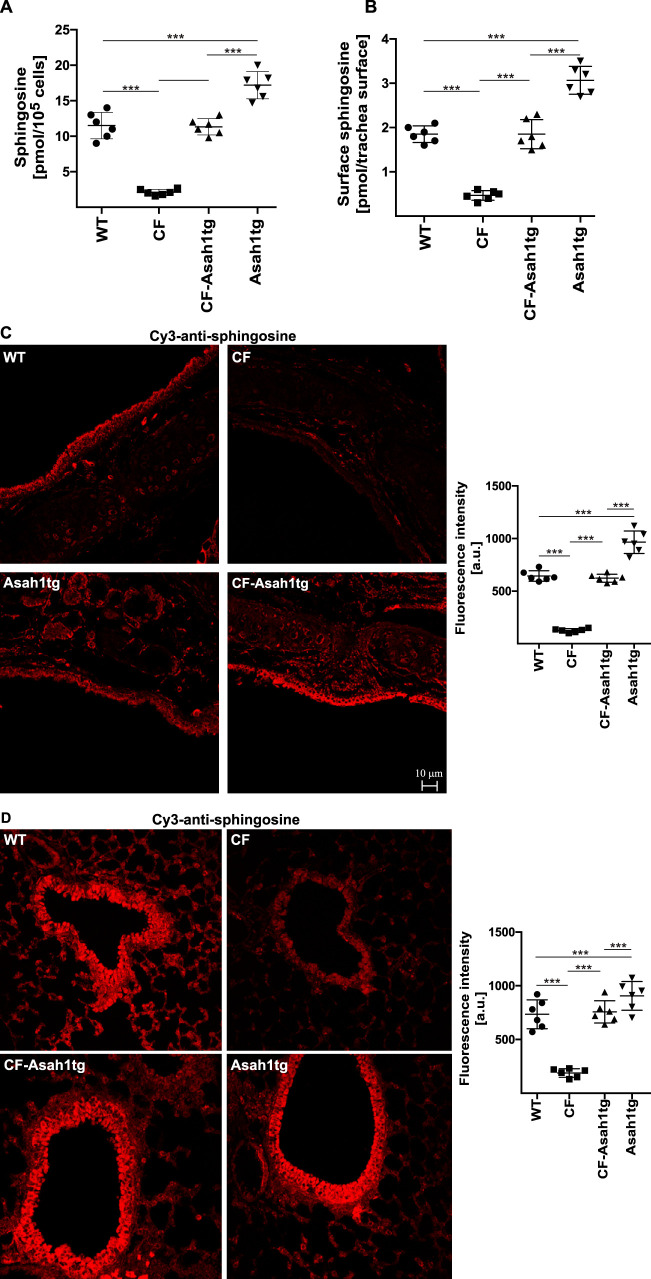
Transgenic expression of acid ceramidase in CF mice restores anti-sphingosine levels in the trachea and lung of CF mice. Sphingosine levels in the trachea and lung of syngeneic wild-type, CF, Asah1tg, and CF-Asah1tg mice were determined by kinase assays on lysates of isolated tracheal epithelial cells (A), *in situ* kinase assays measuring the concentration of anti-sphingosine in the luminal membrane of tracheal epithelial cells (B), and confocal fluorescence microscopy of anti-sphingosine immunostaining of trachea (C) and lung (D) sections. The results demonstrate that the transgenic expression of acid ceramidase in CF mice restores sphingosine levels in airway epithelial cells of CF mice. Confocal microscopy studies were performed with Cy3-coupled antisphingosine antibodies. Given are the means ± SD or a representative result from 6 independent studies each. ***, *P* < 0.001 (by ANOVA and *post hoc* Student’s *t* test).

Acid ceramidase generates sphingosine from ceramide. Sphingosine levels are decreased in CF epithelial cells (11–13). We therefore determined the concentration of sphingosine in isolated tracheal epithelial cells from wild-type, CF, and CF-Asah1tg mice. The results confirm the reduction of sphingosine in CF mice compared to wild-type mice (Fig. 2A). The transgenic overexpression of acid ceramidase in CF mice restored sphingosine levels in the trachea of these mice compared to CF mice (Fig. 2A). Sphingosine levels in CF-Asah1tg mice did not differ from sphingosine levels in control wild-type mice. *In situ* kinase assays that allow quantitative measurements of surface sphingosine in tracheal epithelial cells also revealed an increase of surface sphingosine in CF-Asah1tg mice compared to CF mice (Fig. 2B). This indicates that the expression of the transgene is added to the expression of endogenous acid ceramidase, which is reduced in CF mice. The data from the biochemical studies were confirmed by confocal fluorescence microscopy studies that also showed a marked increase of sphingosine on the surface of epithelial cells of CF-Asah1tg mice compared to CF mice (Fig. 2C).

**FIG 3:**
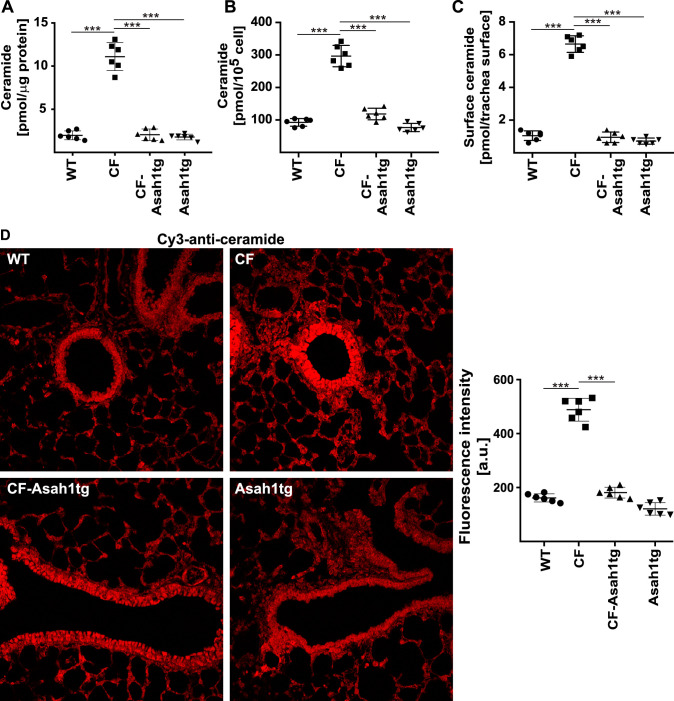
Transgenic expression of acid ceramidase in CF mice normalizes increased ceramide levels in the trachea and lung of CF mice. Ceramide levels in the trachea and lungs of syngeneic wild-type, CF, Asah1tg, and CF-Asah1tg mice were determined by kinase assays on lung lysates (A) and isolated tracheal epithelial cells (B), *in situ* kinase assays measuring the concentration of ceramide in the luminal membrane of tracheal epithelial cells (C), and confocal fluorescence microscopy (D). The results show that the transgenic expression of acid ceramidase in CF mice normalizes the increased ceramide levels in airway epithelial cells of CF mice and reduces the concentration of ceramide in tracheal and bronchial epithelial cells of CF mice to levels that are comparable to the ceramide levels in wild-type mice. Confocal microscopy studies were performed with Cy3-coupled anti-ceramide antibodies. Fluorescence was also quantified (D). Given are the means ± SD or a representative result from 6 independent studies each. ***, *P* < 0.001 (by ANOVA and *post hoc* Student’s *t* test).

Previous studies demonstrated that the application of acid ceramidase to CF mice also normalizes ceramide levels in epithelial cells of the airways. Thus, to further characterize CF-Asah1tg mice, we measured total ceramide in the lungs and tracheas of wild-type, CF, and CF-Asah1tg mice. The results confirm the function of the transgene in CF-Asah1tg mice and demonstrate that the transgenic expression of Asah1 normalized ceramide levels compared to CF mice (Fig. 3A and B). CF mice showed increased levels of ceramide in the lung and trachea (Fig. 3A and B). Likewise, we determined surface ceramide by *in situ* kinase assays of the tracheal surface and detected that the transgenic expression of the acid ceramidase also normalized ceramide on the surface of tracheal epithelial cells (Fig. 3C). Ceramide levels were increased in CF mice compared to wild-type mice. The effect of the transgenic expression of acid ceramidase on ceramide levels in airway epithelial cells was also demonstrated in confocal microscopy studies that revealed the normalization of ceramide specifically in bronchial epithelial cells of CF-Asah1tg mice compared to wild-type and CF mice (Fig. 3D).

**FIG 4:**
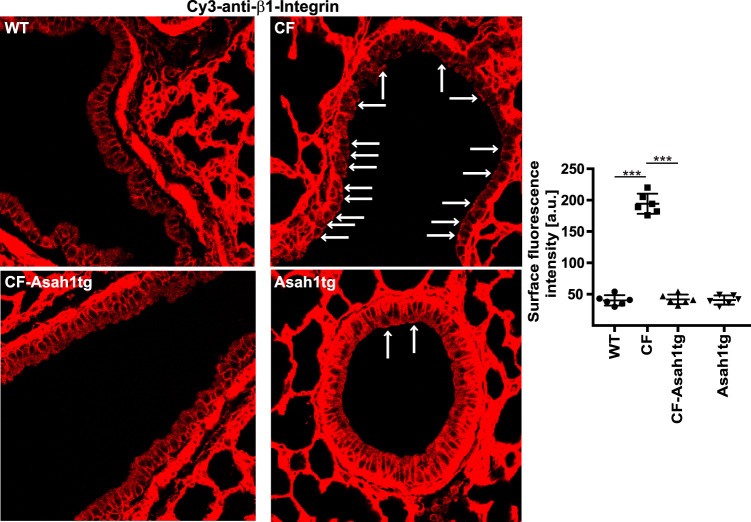
Transgenic expression of acid ceramidase in CF mice abrogates ectopic surface expression of β1-integrin in CF mice. Confocal microscopy studies reveal that β1-integrin is absent from the luminal membrane of the bronchial epithelial cell surface in wild-type or Asah1tg mice, while CF mice accumulate β1-integrin in the luminal membrane of bronchial epithelial cells. The transgenic expression of acid ceramidase in CF-Asah1tg mice normalizes the expression of β1-integrin and abrogates its ectopic expression. The confocal microscopy studies were performed with Cy3-coupled anti-β1-integrin antibodies. Displayed are representative results from 6 independent studies each. Surface β1-integrin is indicated by arrows. Fluorescence was also quantified. The means ± SD are shown (*n* = 6). ***, *P* < 0.001 (by ANOVA and *post hoc* Student’s *t* test).

We have shown that increased ceramide levels result in the ectopic expression of β1-integrin on the surface of bronchial epithelial cells in CF airways, while β1-integrin is absent from the luminal surface of wild-type airways (13). The ectopic expression of β1-integrin is critically important for the increased susceptibility of CF mice to infections with P. aeruginosa or S. aureus as evidenced in experiments in which we blocked β1-integrin (13). This blockade prevented infections of CF mice with pathogens (13). Thus, we tested whether the transgenic expression of acid ceramidase in CF mice also normalizes the expression of β1-integrin. Confocal microscopy studies demonstrated the ectopic expression of β1-integrin in CF epithelial cell layers, which was abrogated in the epithelial cell layers of CF-Asah1tg mice (Fig. 4). The pattern of β1-integrin expression in these mice corresponded to that in wild-type mice.

**FIG 5:**
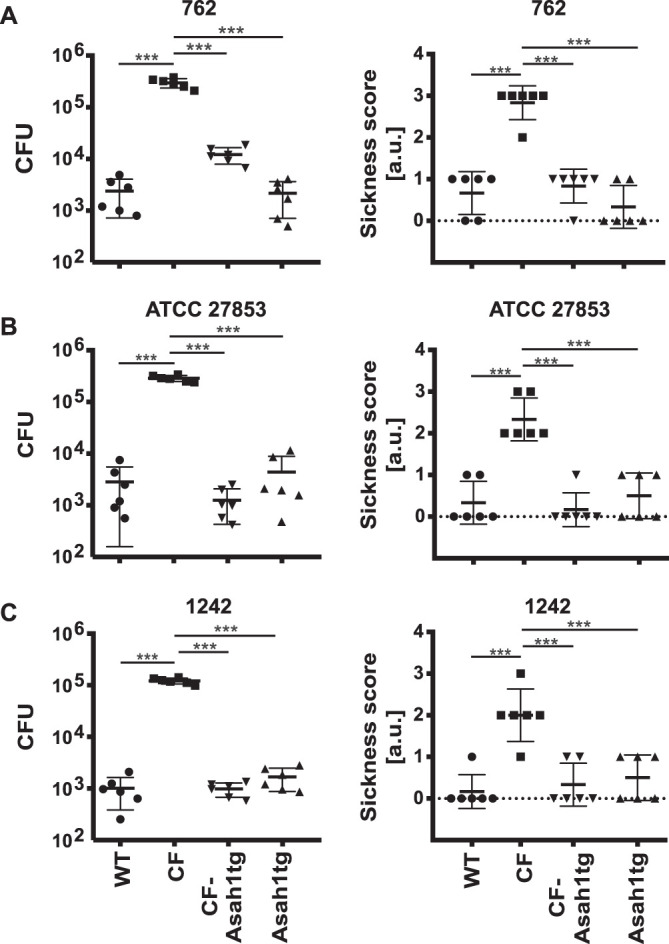
Overexpression of acid ceramidase protects CF mice from pulmonary infection with P. aeruginosa. (A and B) Cystic fibrosis (CF), wild-type (WT), Asah1tg, or CF-Asah1tg mice were infected with P. aeruginosa strains 762 (A), ATCC 27853 (B), and 1242 (C) for 4 h. Pneumonia was determined by using a sickness score and measuring the number of bacteria (CFU) in the lung 4 h after infection. The overexpression of acid ceramidase protected CF mice from pulmonary infection with P. aeruginosa. Shown are the means ± SD from 6 mice each. ***, *P* < 0.001 (by ANOVA and *post hoc* Student’s *t* test).

Acute and chronic infections with P. aeruginosa and S. aureus are some of the most important problems of CF patients (1, 21, 22). Thus, we determined the infection susceptibility of CF-Asah1tg mice. To this end, we infected wild-type, CF, Asah1tg, and CF-Asah1tg mice intranasally with three different P. aeruginosa strains, a septic, a laboratory, and a mucosal one, and determined the number of bacteria in the lungs of these animals and the score of sickness of the mice directly before lung resection. CF mice were very susceptible to the pathogens, and the lungs already contained high numbers of bacteria a few hours after intranasal application, while wild-type and Asah1tg mice were almost completely resistant to infection (Fig. 5A to C). The transgenic overexpression of acid ceramidase in CF-Asah1tg mice restored the resistance of CF mice to P. aeruginosa. Infection resulted in very low numbers of the pathogens in the lungs of these mice, very similar to the bacterial numbers in wild-type mice (Fig. 5A to C), and almost abrogated the clinical symptoms typical of pneumonia (Fig. 5A to C).

**FIG 6:**
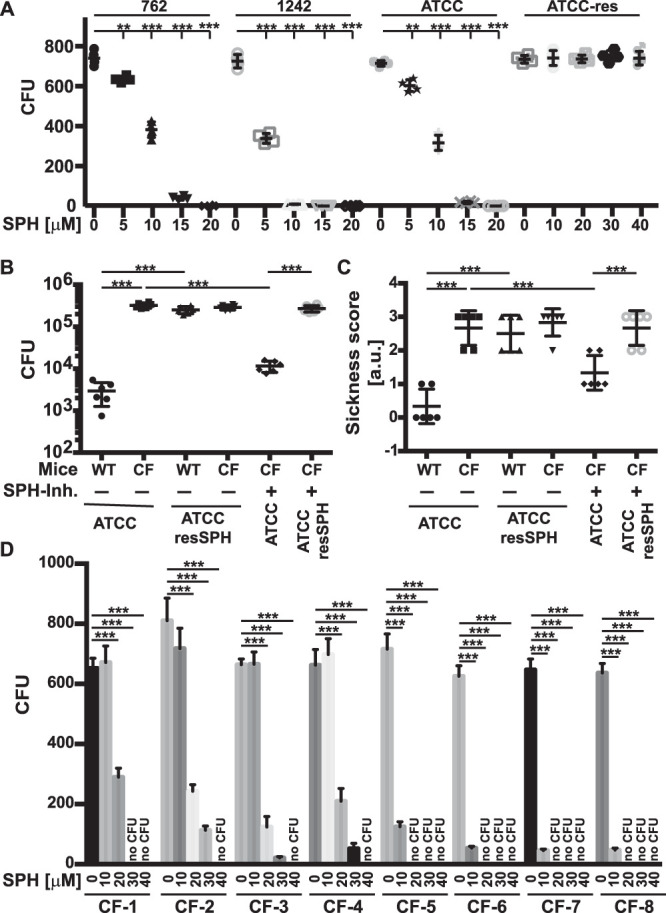
Sphingosine mediates killing of P. aeruginosa. (A) Comparison of the sensitivities of P. aeruginosa strains 762, 1242, ATCC 27853, and the sphingosine-resistant P. aeruginosa ATCC 27853 strain (ATCC 27853resSPH [ATCC-res]). A total of 1,000 CFU were incubated in H/S for 60 min with increasing concentrations of sphingosine, aliquots were plated on LB agar, and CFU were counted after growth overnight. Shown are the means ± SD from 4 independent experiments. **, *P* < 0.01; ***, *P* < 0.001 (by ANOVA and *post hoc* Student’s *t* test). (B and C) CF or wild-type (WT) mice were infected with the parental wild-type P. aeruginosa strain ATCC 27853 or the sphingosine-resistant mutant of P. aeruginosa strain ATCC 27853 (ATCC 27853resSPH). In addition, CF mice inhaled with 125 μM sphingosine (SPH-Inh.) for 10 min or 30 min prior to infection. Pneumonia was determined by measuring the number of the bacteria (CFU) in the lung 4 h after infection (B) and by employing a sickness score (C). Shown are the means ± SD from 5 mice each. ***, *P* < 0.001 (by ANOVA and *post hoc* Student’s *t* test). (D) Analysis of a panel of clinical P. aeruginosa isolates CF1 to CF8 from CF patients shows that all strains were sensitive to sphingosine and were killed at between 10 μM and 40 μM sphingosine. Displayed are the means ± SD from 6 independent experiments each measuring the number of CFU with or without sphingosine. **, *P* < 00.1; ***, *P* < 0.001 (by ANOVA and *post hoc* Student’s *t* test). Values were tested for normal distribution.

The product of the activity of acid ceramidase is sphingosine. Thus, we aimed to determine whether sphingosine or a metabolite mediates the killing of P. aeruginosa in the lung. To this end, we generated a P. aeruginosa clone from ATCC 27853 that is resistant to sphingosine. This was achieved by repeated incubation with 40 μM sphingosine. We obtained 5 different clones and used one of these clones in the present studies. A dose-response curve with sphingosine shows that the strain exhibits high-level resistance to sphingosine (Fig. 6A). We infected wild-type mice that had high concentrations of sphingosine in their airways (13) with the wild-type ATCC 27853 and sphingosine-resistant (ATCC 27853resSPH) strains and determined the development of pneumonia. Wild-type mice rapidly cleared the wild-type ATCC 27853 strain from the lung, while they developed severe pneumonia upon infection with the sphingosine-resistant strain (Fig. 6B). Next, we infected CF mice and CF mice that were treated by sphingosine inhalation prior to infection with wild-type P. aeruginosa ATCC 27853 and the sphingosine-resistant P. aeruginosa strain. Cystic fibrosis mice should be sensitive to both strains because sphingosine concentrations in their airways are low. If sphingosine is important for defense against P. aeruginosa, inhalation of sphingosine in CF mice should rescue these mice from infection with wild-type P. aeruginosa strain ATCC 27853, while inhalation should have no or a reduced effect on infection with the sphingosine-resistant strain. If a metabolite of sphingosine mediates the killing of P. aeruginosa in the lung, inhalation of sphingosine should have comparable effects on both strains.

The results showed that infection of CF mice with P. aeruginosa wild-type strain ATCC 27853 or the sphingosine-resistant strain ATCC 27853resSPH resulted in severe pulmonary infection (Fig. 6C). Inhalation of sphingosine rescued CF mice from infection with P. aeruginosa wild-type strain ATCC 27853. In contrast, and most importantly, inhalation of sphingosine did not rescue CF mice from infection with the sphingosine-resistant strain (Fig. 6C).

To test whether patients with cystic fibrosis are infected with P. aeruginosa strains that are sensitive or resistant to sphingosine, we tested the sensitivities of a panel of mucoid and nonmucoid P. aeruginosa strains isolated from CF patients. All of these strains were sensitive to sphingosine (Fig. 6D), although all of them showed resistance to many clinically used antibiotics (Table 1).

**TABLE 1:** Profiles of resistance of clinical isolates against antibiotics^*a*^

Antibiotic	Resistance profile of isolate (material, type of growth)						
	CF-1 (sputum, nonmucoid)	CF-2 (sputum, mucoid)	CF-3 (sputum, mucoid)	CF-4 (throat swab, nonmucoid)	CF-5 (sputum, mucoid)	CF-6 (throat swab, nonmucoid)	CF-8 (sputum, mucoid)
Amoxicillin	R	R	R	R	R	R	R
Amoxicillin-clavulanic acid	R	R	R	R	R	R	R
Ampicillin	R	R	R	R	R	R	R
Ampicillin-sulbactam	R	R	R	R	R	R	R
Piperacillin	I	I	I	I	R	I	I
Piperacillin-tazobactam	I	I	I	I	R	I	I
Cefotaxime	R	R	R	R	R	R	R
Ceftriaxone	R	R	R	R	R	R	R
Ceftazidime	I	I	I	I	R	I	I
Imipenem	I	R	I	I	R	I	I
Meropenem	R	I	S	S	I	S	R
Tobramycin	S	R	S	S	R	S	S
Amikacin	R	R	S	S	R	S	R
Co-trimoxazole	R	R	R	R	R	R	R
Ciprofloxacin	R	I	I	I	I	I	R
Levofloxacin	R	I	I	I	I	I	R
Nitrofurantoin	R	R	R	R	R	R	R
Colistin	S	S	S	S	S	S	S

a The resistance of the clinical isolates to most clinically employed antibiotics was determined as described in the text. R, resistant; S, sensitive; I, susceptible after exposure to increased concentrations.

## DISCUSSION

Here, we demonstrate that the transgenic overexpression of acid ceramidase in CF mice is sufficient not only to normalize ceramide and sphingosine levels but also to normalize functional consequences such as the ectopic expression of β1-integrin and, most importantly, to restore resistance to pulmonary P. aeruginosa infections. These studies further support a key role of acid ceramidase and sphingosine in the pathophysiology of CF. Studies employing a sphingosine-resistant P. aeruginosa strain support the notion that sphingosine is key to the pulmonary elimination of P. aeruginosa and that this defense mechanism is defective in CF mice.

We have previously shown the reduced expression of acid ceramidase in tracheal and bronchial epithelial cells of CF mice but also in the lungs of CF patients and in liquid airway cultures of human epithelial cells (11–13). We also demonstrated that the application of recombinant acid ceramidase normalized ceramide and sphingosine levels and prevented infections with P. aeruginosa and S. aureus. However, these studies used a pharmacological approach applying acid ceramidase exogenously and do not prove that endogenous acid ceramidase is important for resistance to bacterial pneumonia. Thus, we aimed to provide genetic proof of the protective role of endogenous acid ceramidase in bacterial infections of the airways.

The transgenic overexpression of acid ceramidase results in the normalization of sphingosine levels in respiratory epithelial cells. Sphingosine kills bacterial pathogens on epithelial surfaces and the skin (11–13, 17–20). We have recently shown that sphingosine binds to cardiolipin, clusters the lipid, and induces the formation of large and very rigid domains (20). The alterations of the biophysical properties of the plasma membrane result in leakiness of the plasma membrane, rapid depolarization, and killing of the bacteria (20). Since sphingosine must directly contact and even enter the bacteria, it seems to be important that the overexpression of acid ceramidase restores the surface levels of sphingosine, which comes into direct contact with invading pathogens. In addition, cells might release microparticles containing sphingosine, and the sphingosine concentration in these particles very likely also depends on the expression levels of acid ceramidase.

At present, it is unknown how acid ceramidase is downregulated in CF cells. To overcome this gap, we used the approach of overexpressing acid ceramidase. In this approach, acid ceramidase expression is controlled by the CAG promoter and does not underlie the endogenous regulation of the expression of acid ceramidase.

Previous studies have indicated that inhalation of recombinant acid ceramidase normalizes the expression of ceramide, sphingosine, and β1-integrin and, most importantly, also infection with P. aeruginosa. The present study demonstrates that normalization of the endogenous expression of acid ceramidase in CF airways also normalizes these pathologies and protects CF mice from severe pulmonary infection with P. aeruginosa.

Clinically, bacterial lung infections constitute the major problem for most patients with CF (1, 21, 23). At present, gastrointestinal symptoms can be well controlled, but it is still very difficult and often impossible to prevent acute and finally chronic pneumonia. Chronic pneumonia, which is caused by P. aeruginosa in most CF patients, is also often the reason for the progressive destruction of the lung and shortened life expectancy in these patients. Moreover, chronic P. aeruginosa infections are also often observed in patients with chronic obstructive pulmonary disorder (COPD) (22). Ceramide has been shown to be increased in the lungs of COPD patients (24, 25), and COPD might be another disease in which acid ceramidase might be downregulated.

Therefore, collectively, these studies strongly support the notion that treatment of CF patients with acid ceramidase or sphingosine may be very beneficial and may protect patients from the development of pulmonary infections.

## MATERIALS AND METHODS

### Mice.

B6.129P2(CF/3)-*Cftr*^TgH(neoim)Hgu^ (CF^MHH^) congenic mice were generated as previously described (4, 26, 27). Briefly, *Cftr*^TgH(neoim)Hgu^ mutant mice, which were generated by insertional mutagenesis in exon 10 of the *Cftr* gene, were inbred to create the congenic *Cftr^MHH^* strain. These mice were then backcrossed for more than 20 generations to a C57BL/6 background. Because these mice still express low levels of Cftr, they can be fed a standard mouse diet. They exhibit normal development but also display pulmonary pathology typical of CF (4–6, 28). Syngeneic C57BL/6 mice were used as wild-type controls.

*Cftr^MHH^* mice were crossed with mice transgenic for Asah1 to overexpress the acid ceramidase in mice lacking Cftr. Asah1 transgenic mice were also on a C57BL/6 background. The overexpression of the acid ceramidase was achieved by introducing an expression cassette in which Asah1 is expressed under the control of the CAG promoter into the mouse genome. Mice were cloned, and the integration of the transgene was tested by PCR. The overexpression of acid ceramidase was confirmed by measuring acid ceramidase activity in several tissues.

Asah1 transgenic (Asah1tg0) mice served as controls.

We used female and male mice at an age of at least 16 weeks and weighing between 25 and 35 g. Mice were divided into cages of equal sizes (usually 3 to 4 mice) by animal unit technical staff with no involvement in study design. Cages were randomly assigned to an experimental group. The investigators were blind to group allocation during the experiment and/or when assessing the outcome.

Mice were housed and bred within isolated and ventilated cages in the mouse facility of the University Hospital, University of Duisburg-Essen, Germany. They were repeatedly tested for a panel of common murine pathogens according to the 2002 recommendations of the Federation of European Laboratory Animal Science Associations (https://felasa.eu/working-groups/guidelines), including S. aureus. The mice were free of all pathogens. Animal procedures were approved by the Bezirksregierung Duesseldorf, Duesseldorf, Germany, and the IACUC committee Cincinnati.

### Immunohistochemistry of mouse trachea and lungs.

Mouse trachea and lungs were stained for acid ceramidase, sphingosine, or ceramide as previously described (11–13). Mice were sacrificed by cervical dislocation, and the trachea and lungs were removed and immediately fixed in 4% phosphate-buffered saline (PBS)-buffered paraformaldehyde (PFA) (catalog number 0335.3; Roth). The trachea was opened prior to fixation. Fixation was performed for 48 h. The tissue was serially dehydrated with an ethanol-to-xylol gradient, embedded in paraffin, sectioned at 7 μm, dewaxed, and rehydrated. To retrieve the antigens, sections were treated with pepsin (Digest All, catalog number 003009; Invitrogen) for 30 min at 37°C. The sections were then washed, blocked for 10 min at room temperature with PBS, supplemented with 5% fetal calf serum (FCS), washed once in PBS, and stained with anti-acid ceramidase (1:100 dilution) (catalog number 4741; ProSci, Heidelberg, Germany), anti-ceramide (1:200 dilution) (clone S58-9, catalog number MAB_0011; Glycobiotech), anti-sphingosine (1:1,000 dilution) (clone NHSPH, catalog number ALF-274042010; Alfresa Pharma Corporation, Osaka, Japan), or anti-mouse β1-integrin (clone MB1.2, catalog number MAB1997; Merck Millipore) antibody at room temperature for 45 min. Antibodies were diluted in HEPES/Saline (H/S) consisting of 132 mM NaCl, 20 mM HEPES [pH 7.4], 5 mM KCl, 1 mM CaCl_2_, 0.7 mM MgCl_2_, 0.8 mM MgSO_4_ plus 1% FCS. The sections were washed three times for 5 min each with PBS plus 0.05% Tween 20 and once with PBS. The tissues were then secondarily labeled with Cy3-coupled anti-rabbit IgG (for anti-acid ceramidase antibodies), Cy3-coupled anti-mouse IgM (for anti-sphingosine or anti-ceramide antibodies), or anti-rat IgG (for anti-β1-integrin antibodies) F(ab)_2_ fragments (Jackson Immunoresearch) in H/S plus 1% FCS for 30 min. Sections were washed three times as described above and once with PBS and embedded in Mowiol. Samples were evaluated by confocal microscopy on a Leica TCS-SL confocal microscope equipped with a 40× lens, and images were analyzed with Leica LCS software (Leica Microsystems, Mannheim, Germany). All comparative samples were measured at identical settings. Control stainings were performed with Cy3-labeled secondary antibodies only or with irrelevant rabbit IgG, mouse IgM, or rat IgG, followed by staining with the corresponding Cy3-labeled secondary antibodies. These controls revealed very weak staining and confirmed the specificity of the antibody staining. Fluorescence intensities were quantified using ImageJ.

### Surface activity of acid ceramidase.

Mice were sacrificed; the trachea was removed, opened, and placed on a 37°C prewarmed plastic plate; and 4 μl of a buffer consisting of 150 mM sodium acetate (pH 7.4), 0.05% octylglucopyranoside, and [^14^C_16_]ceramide (55 mCi/mmol) (catalog number ARC-0831; ARC, St. Louis, MO, USA) was pipetted onto the lumen of the trachea. Prior to the assay, [^14^C_16_]ceramide was dried, resuspended, and bath sonicated for 10 min. The assay was performed for 15 min, and the trachea was then extracted in H_2_O and CHCl_3_-CH_3_OH-HCl (100:100:1, vol/vol/vol). The lower phase was dried, resuspended in CHCl_3_-CH_3_OH (1:1, vol/vol), separated by thin-layer chromatography (TLC) with CHCl_3_-CH_3_OH-NH_4_OH (90:20:0.5, vol/vol/vol), and analyzed with a Fuji phosphorimager.

### Luminal surface levels of ceramide.

The intact epithelial surface of the trachea was incubated with 0.01 U diacylglycerol (DAG) kinase (catalog number BML-SE100; Enzo) in a solution containing 4 μl of 150 mM sodium acetate (pH 7.4), 1 mM ATP, and 10 μCi [γ-^32^P]ATP for 15 min at 30°C. Controls were incubated with the same buffer without kinase or were left untreated. The kinase reaction was terminated by transferring the trachea into a solution containing CHCl_3_–CH_3_OH–1 N HCl (100:100:1, vol/vol/vol), 170 μl of a buffered saline solution (135 mM NaCl, 1.5 mM CaCl_2_, 0.5 mM MgCl_2_, 5.6 mM glucose, 10 mM HEPES [pH 7.2]) and 30 μl of 100 mM EDTA were added, and lipids were extracted, separated on silica G60 TLC plates, and analyzed by liquid scintillation counting and a standard curve of C_16_- to C_24_-ceramides, as previously described (11, 13).

### Luminal surface levels of sphingosine.

Mice were sacrificed, and the trachea was removed, opened, and placed on a 30°C prewarmed plastic plate. We then added 4 μl of kinase buffer consisting of 150 mM sodium acetate (pH 7.4), 0.001 U of sphingosine kinase 1 (catalog number 6068-SK-010; R&D, Minneapolis, MN, USA), 1 μM ATP, and 10 μCi [γ-^32^P]ATP onto the luminal surface of the trachea. Controls were incubated with the same buffer without sphingosine kinase. The kinase reaction was terminated by the addition of the trachea to 100 μl H_2_O, followed by the addition of a solution containing 20 μl 1 N HCl, 800 μl CHCl_3_–CH_3_OH–1 N HCl (100:200:1, vol/vol/vol), and 240 μl each of CHCl_3_ and 2 M KCl. Phases were separated, and the lower phase was collected, dried, dissolved in 20 μl CHCl_3_-CH_3_OH (1:1, vol/vol), and separated on silica G60 TLC plates with CHCl_3_-CH_3_OH-acetic acid-H_2_O (90:90:15:5, vol/vol/vol/vol) as a developing solvent. The TLC plates were analyzed with a phosphorimager. Surface sphingosine levels were determined with a standard curve of C_18_-sphingosine.

### Acid ceramidase activity in trachea extracts.

Tracheae were shock frozen, lysed in 1% Igepal in 150 mM sodium acetate (pH 4.5), and immediately sonicated with a tip sonicator to contain homogeneous lysates. Samples were diluted in 0.1% NP-40 in 150 mM sodium acetate (pH 4.5), and 0.3 μCi/sample [^14^C_16_]ceramide (catalog number ARC-0831) (55 mCi/mmol) was added. To this end, [^14^C_16_]ceramide was dried for 10 min, resuspended in 0.1% octylglucopyranoside (OGP) in 150 mM sodium acetate (pH 4.5), and bath sonicated for 10 min. The samples were incubated at 37°C for 60 min. The reaction was terminated by extraction in H_2_O and CHCl_3_-CH_3_OH-HCl (100:100:1, vol/vol/vol). The lower phase was dried, and samples were resuspended in CHCl_3_-CH_3_OH (1:1, vol/vol), separated by TLC with CHCl_3_-CH_3_OH-NH_4_OH (90:20:0.5, vol/vol/vol), and analyzed with a Fuji imager.

### Ceramide and sphingosine measurements in isolated tracheal epithelial cells and trachea and lung extracts.

Tracheae or lungs were isolated, and epithelial cells were carefully removed from the surface and lysed in a solution containing 1% Igepal, 25 mM Tris-HCl (pH 7.4), 125 mM NaCl, 10 mM EDTA, 10 mM sodium pyrophosphate, and 10 μg/ml each aprotinin and leupeptin. Aliquots were extracted in CHCl_3_–CH_3_OH–1 N HCl (100:200:1, vol/vol/vol). The lower phase was dried and resuspended in a detergent solution consisting of 7.5% (wt/vol) *n*-octyl glucopyranoside and 5 mM cardiolipin in 1 mM diethylenetriamine-pentaacetic acid (DTPA). The kinase reaction was initiated by the addition of 0.001 U sphingosine kinase in a solution containing 50 mM HEPES (pH 7.4), 250 mM NaCl, 30 mM MgCl_2_, 1 mM ATP, and 10 μCi [γ-^32^P]ATP or 10 μl DAG kinase (GE Healthcare Europe, Munich, Germany), 0.1 M imidazole-HCl (pH 6.6), 0.2 mM DTPA, 70 mM NaCl, 17 mM MgCl_2_, 1.4 mM ethylene glycol tetraacetic acid, 2 mM dithiothreitol, 1 μM ATP, and 5 μCi [γ-^32^P]ATP. Samples were incubated for 60 min at 37°C with shaking (350 rpm) and processed as described above.

### Ceramide measurements in lung extracts.

Tracheae or lungs were shock frozen; lysed in a solution containing 1% Igepal, 25 mM Tris-HCl (pH 7.4), 125 mM NaCl, 10 mM EDTA, 10 mM sodium pyrophosphate, and 10 μg/ml each aprotinin and leupeptin; and immediately sonicated with a tip sonicator to contain homogeneous lysates. Aliquots were extracted in CHCl_3_–CH_3_OH–1 N HCl (100:200:1, vol/vol/vol). The lower phase was dried and resuspended in a detergent solution (7.5% [wt/vol] *n*-octyl glucopyranoside and 5 mM cardiolipin in 1 mM DTPA). The kinase reaction was initiated by the addition of a solution containing 10 μl DAG kinase (GE Healthcare Europe, Munich, Germany), 0.1 M imidazole-HCl (pH 6.6), 0.2 mM DTPA, 70 mM NaCl, 17 mM MgCl_2_, 1.4 mM EGTA, 2 mM dithiothreitol, 1 μM ATP, and 5 μCi [γ-^32^P]ATP. Samples were incubated for 60 min at 37°C with shaking (350 rpm) and processed as described above.

### Infections.

P. aeruginosa strain 762, ATCC 27858, a mucoid isolate from individuals with CF, P. aeruginosa strain 1242, or the sphingosine-resistant P. aeruginosa strain was grown overnight on tryptic soy agar (TSA) plates and resuspended in 40 ml prewarmed, sterile tryptic soy broth (TSB) (Becton, Dickinson Biosciences) in Erlenmeyer flasks at an optical density (OD) of 0.200 to 0.250. Bacteria were then grown in TSB for 60 min at 37°C with shaking at 125 rpm until they reached the early logarithmic phase. After this, bacteria were centrifuged (10 min at 1,710 × *g*), the supernatant was removed, bacteria were washed with prewarmed RPMI 1640 plus 10 mM HEPES and resuspended in the same medium, and the concentration was determined by measurement of the OD and comparison to a calibration curve. Finally, the concentration of the bacteria was adjusted to 5 × 10^6^ CFU per 20 μl in prewarmed RPMI 1640 medium plus 10 mM HEPES (4, 13), and the inoculum was directly used (4, 13). If indicated, mice inhaled 800 μl of a 125 μM sphingosine suspension in 0.9% NaCl 30 min prior to infection (the mice inhale less than 10% of the inhalation volume). To infect mice with the P. aeruginosa strains, mice were lightly anesthetized as previously described (4, 29). The mice were then inoculated intranasally with 5 × 10^6^ CFU P. aeruginosa by employing a plastic-coated 30-gauge needle, which was inserted 2 mm into the nose. We determined the mouse health status 4 h after infection using the following scores: a sickness score of 0 for unaffected (healthy appearance), a score of 1 for slightly affected (ruffled fur), a score of 2 for moderately affected (ruffled fur, breathing slightly impaired, and normal body temperature), and a score of 3 for severely affected (ruffled fur, heavy breathing, and lower body temperature). Bacterial numbers were then determined in the mouse lungs 4 h after infection. To this end, mice were sacrificed, and the lungs were removed, homogenized, and lysed in 5 mg/ml saponin to release intracellular bacteria. Bacteria were then pelleted at 2,240 × *g*, washed once in sterile PBS, diluted, plated, and grown in duplicate on TSA plates for 12 h. Bacterial numbers were counted. Infection experiments were approved by the Bezirksregierung Duesseldorf, Duesseldorf, Germany, under permission numbers 84-02.04.2013.A348 and 81-02.04.2019.A134 and by the IACUC committee Cincinnati. The actual care and treatment of the animals were performed and/or overseen by veterinarians of the Central Animal Facility of the University Hospital Essen, Essen, Germany.

### Sphingosine treatment of P. aeruginosa *in vitro*.

To test the antibacterial efficacy of sphingosine in killing different P. aeruginosa strains *in vitro*, we incubated 1 × 10^3^ bacteria in H/S with increasing amounts of sphingosine (C_18_-sphingosine; Avanti Polar Lipids, Inc., AL, USA). To this end, a sphingosine stock solution of 10 mM in 0.9% NaCl was prepared by sonication and stored at −20°C. The stock solution was bath sonicated directly before use, diluted in H/S, and sonicated again for 10 min. Bacteria were incubated with the indicated sphingosine concentrations for 1 h at 37°C on a horizontal shaker at 125 rpm. After this time, an aliquot of the bacteria was plated on Luria broth (LB) agar plates and incubated overnight to determine bacterial survival.

### Generation of sphingosine-resistant P. aeruginosa strains.

P. aeruginosa strain ATCC 27853 was prepared as described above, and 24 aliquots of 1,000 CFU each were incubated for 60 min with 40 μM sphingosine in 500 μl as described above. We then added 500 μl TSB, and cultures were grown overnight. Resistant cultures were plated on TSB agar plates, and clones were picked after growth overnight. Clones were treated for 5 additional rounds with 40 μM sphingosine and then used for experiments.

### Clinical P. aeruginosa strains and antibiotic resistance measurements.

All clinical samples were used after performing conventional microbiological diagnostics. The study did not include patients’ details and did not result in additional constraints for the patients. All data were anonymously analyzed without the need for patient consent due to the retrospective nature of the study. All procedures and methods were carried out in accordance with approved guidelines.

In this study, eight clinical Pseudomonas aeruginosa strains were included (Table 1). All isolates were derived from sputum samples or throat swabs from CF patients. Antibiotic susceptibility testing was performed with commercial methods (MicroScan WalkAway plus, panel NC83; Beckman Coulter, Brea, CA, USA). Susceptibility testing was performed according to the manufacturer’s instructions. For interpretation, EUCAST breakpoints (version 10.0, 2020) were used.

### Quantification and statistical analysis.

Data are expressed as arithmetic means ± standard deviations (SD). For the comparison of continuous variables from independent groups, we used Student’s *t* test for two groups and one-way analysis of variance (ANOVA) for more than two groups followed by *post hoc* Student’s *t* test for all pairwise comparisons applying Bonferroni correction for multiple testing. The *P* values for the pairwise comparisons were calculated after Bonferroni correction. All values were normally distributed. The statistical details (numbers, means ± SD, and tests) are given in the figure legends. The sample size planning for the continuous variables in *in vivo* infection experiments was based on two-sided Wilcoxon-Mann-Whitney tests (G*Power version 3.1.7 software of the University of Duesseldorf, Germany). Investigators were blind for histology experiments and animal identity as described above. All data were quantified using ImageJ and are expressed as arbitrary units. In each photo, 20 randomly chosen areas corresponding to the luminal membrane of 20 different cells were quantified and averaged with the values obtained in the other photos from the fluorescence microscopy studies. G*Power version 3.1.7 of the University of Duesseldorf, Germany, and ImageJ are public programs available online without charge.
